# Hypertension and Cardiovascular Diseases: From Etiopathogenesis to Potential Therapeutic Targets

**DOI:** 10.3390/ijms23147742

**Published:** 2022-07-13

**Authors:** Iveta Bernatova, Silvia Liskova, Monika Bartekova

**Affiliations:** 1 Institute of Normal and Pathological Physiology, Centre of Experimental Medicine, Slovak Academy of Sciences, Sienkiewiczova 1, 81371 Bratislava, Slovakia; iveta.bernatova@savba.sk; 2 Institute of Pharmacology and Clinical Pharmacology, Faculty of Medicine, Comenius University, Sasinkova 4, 81108 Bratislava, Slovakia; 3 Institute for Heart Research, Centre of Experimental Medicine, Slovak Academy of Sciences, 9 Dúbravská Cesta, 84104 Bratislava, Slovakia

Cardiovascular diseases (CVDs) are the top cause of death worldwide, and arterial hypertension per se remains the major preventable cause of CVDs. Risk factors of CVDs consist of several non-modifiable factors (genetic composition, age, sex, race) and modifiable factors (such as smoking, diet-related factors, sedentary lifestyle), and stress. CVDs are often associated with dyslipidemia, type two diabetes mellitus, hypercholesterolemia, and obesity or renal diseases.

This Special Issue published in *IJMS*, entitled “Hypertension and Cardiovascular Diseases: From Etiopathogenesis to Potential Therapeutic Targets”, aims to highlight the current knowledge in the field of CV research and to stimulate further progress. Twelve contributions have been selected for publication, including pre-clinical research articles, clinical studies and a review, which were preferably associated with molecular sciences. In addition, this Special Issue was jointly organized with *Biomedicines*, in which another eleven articles, presenting more experimental and clinical content, can be found.

The review article by Pena et al. [1] summarizes the relationship between oxidative stress, protein kinase activation and inflammatory implications in right ventricular hypertrophy and heart failure under hypobaric hypoxia. Several mechanisms are able to compensate the hypoxia-induced decrease in oxygen bioavailability including pulmonary artery vasoconstriction and subsequent pulmonary arterial remodeling. These changes can lead to pulmonary hypertension and to the development of right ventricular hypertrophy, right heart failure and, ultimately, to death. The current review describes the most recent molecular pathways involved in the above conditions under hypobaric hypoxia, including oxidative stress, inflammation, protein kinase activation and fibrosis, and the current therapeutic approaches for hypobaric hypoxia. This review also highlights the signaling pathways related to oxidative stress, protein kinase activation, and induction of inflammatory molecules and hypoxic factors, and summarizes recent therapeutic approaches focused on abolishing hypoxia-induced right ventricular hypertrophy and right heart failure. In another article, Pena et al. [2] investigated the expression of nicotinamide adenine dinucleotide phosphate oxidase (Nox) isoforms Nox2 and Nox4 (Nox2/4), p38α mitogen-activated protein kinases (p38αMAPK) and protein kinase B (Akt), hypoxia-inducible factor-1α (HIF-1α) and lectin-like oxidized low-density lipoprotein receptor-1 (LOX-1) in rats exposed to long-term chronic intermittent hypobaric hypoxia (CIH). The authors found increased expression of LOX-1 and Nox2 in right ventricular hypertrophy induced by chronic CIH confirming the relation between the strongly increased levels of nicotinamide adenine dinucleotide phosphate oxidase and LOX-1. Novelty in the knowledge on right ventricular hypertrophy is that in CIH-induced right ventricular hypertrophy the Nox2 isoform expression and its essential component, p22phox, is increased, but there was no change in Nox4 expression. The results of their study suggest that the increase in levels of malondialdehyde, representing lipid peroxidation, were triggered rather by hydroxyl radicals than H_2_O_2_ and via the increase in p38α MAPK activity. Molecular changes in left ventricular hypertrophy were investigated in study by Perera-Gonzalez et al. [3]. The authors focused on the importance of tenascin-C (TN-C) in activation and preservation of the profibrotic stimulus under conditions of pressure overload in wild-type and TN-C knockout (KO) mice. In experimental conditions, the progression of left ventricular hypertrophy under pressure overload significantly reduced ejection fraction in wild-type mice, and the increase in lung weight suggests pulmonary congestion and state of decompensation. De-banding of failing hearts of the wild-type mice led to reverse remodeling and partial reduction in angiotensin-converting enzyme 1 activity and atrial natriuretic protein and collagen 1 expression. TN-C KO mice did not show a significant reduction in left ventricular ejection fraction nor dilatation of the left and right ventricles; thus, addressing TN-C in left ventricular hypertrophy may open a new window for future therapeutics. In the study of Zhang et al. [4], the authors revealed immune-related long non-coding RNAs (lncRNAs) during myocardial infarction in the presence and absence of histidine decarboxylase (HDC), a histamine-synthesizing enzyme involved in the stress response and heart remodeling after myocardial infarction. For this purpose, authors used analysis of immune-associated co-expression networks. Using an acute myocardial infarction mouse model in Hdc-deficient (Hdc−/−) mice, the authors explored the potential roles of Hdc/histamine in cardiac immune responses. Differentially expressed gene (DEG) analysis identified 2126 DEGs in Hdc-deficient groups and 1013 in histamine-treated groups. In addition, the study indicated that myeloid cells and T memory/follicular helper cells were tightly regulated by Hdc/histamine post-myocardial infarction. Finally, four lncRNAs were verified to be closely implicated in tuning the immune responses after myocardial infarction. In conclusion, the study highlighted the HDC-regulated myeloid cells as a driving force contributing to the government of transmission from innate immunocytes to adaptive immunocytes in the progression of the injury response after myocardial infarction, as well as identified the potential role of the Hdc/histamine-lncRNAs network in regulating cardiac immune responses, which may provide novel promising therapeutic targets for further promoting the treatment of ischemic heart disease. The study of Zeng et al. [5] focused on the sex-specific role of endothelial sirtuin 3 (SIRT3) in regulating blood pressure and diastolic function using the endothelial-specific SIRT3 KO mice (SIRT3 ECKO) in which obesity was induced by a high-fat diet. The authors demonstrate that the ablation of endothelial SIRT3 in female mice elevated blood pressure and altered diastolic function as compared with control female mice as well as with male SIRT3 ECKO mice. In addition, female SIRT3 ECKO mice treated with high-fat diet for 20 weeks had an impaired coronary flow reserve and diastolic dysfunction as compared to high-fat-diet-treated male SIRT3 ECKO mice. This study showed a sex-specific role of endothelial SIRT3 in regulating blood pressure and diastolic function in mice, and deficiency of endothelial SIRT3 may be responsible for a diastolic dysfunction in aging females. The study of Zeng et al. also provides a novel model of studying sex differences on hypertension, cardiac hypertrophy, and diastolic dysfunction in mice. In the next study, possible novel treatment options were evaluated under the condition of acute myocardial infarction by Gömöri et al. [6]. Matrix metalloproteinase (MMP)-2 degrades myocardial contractile proteins, as well as sarcoplasmic/endoplasmic reticulum Ca^2+^ ATPase 2a during myocardial ischemia/reperfusion injury. Novel MMP inhibitors (MMPI), MMPI-1154 and MMPI-1260, showed a dose-dependent cardioprotective effect in an in vivo rat model of acute myocardial infarction when administered before reperfusion. However, in the presence of hypercholesterolemia, their infarct size-limiting effect was diminished. This may be explained by the increased MMP-2 activity that correlates positively with serum total and LDL cholesterol levels. Taken together, concomitant administration of MMPI and statins appears to be a promising therapeutic approach for future studies. The MMPs were investigated in the following research article by Kollarova et al. [7]. The authors found elevated levels and activities of MMP-2 in hypertension-induced vascular remodeling. Interestingly, during ageing, the activity of MMP-2 decreases while the MMP-9 activity increases. The authors conclude that the MMP-2 inhibition might be therapeutically applicable during the development of hypertension, while once the hypertension is developed, the systemic MMP-2 and MMP-9 inhibition may not be desirable. The study of Jelemenský et al. [8] evaluated the possible role of extracellular vesicles and/or soluble factors in potential cardioprotective effects of helium conditioning (HeC) in neonatal rat cardiac fibroblasts (NRCFs). To test the hypothesis that HeC induces fibroblast-mediated cardioprotection via extracellular vesicles, isolated NRCFs were exposed to glucose deprivation and HeC followed by a return to normoxic conditions. In addition, the supernatant from HeC-treated NRCFs was transferred to naive NRCFs or immortalized human umbilical vein endothelial cells (HUVEC-TERT2) to test cell migration and angiogenesis. It was found that HeC accelerated the migration of NRCFs but did not increase the expression of fibroblast activation markers. HeC tended to decrease medium extracellular vesicle secretion of NRCFs, but the supernatant of HeC or the control NRCFs did not accelerate the migration of naive NRCFs or affect the angiogenic potential of HUVEC-TERT2. The authors conclude that HeC may contribute to cardioprotection by increasing fibroblast migration, but not by releasing protective medium extracellular vesicles or soluble factors from cardiac fibroblasts. Lichý et al. [9] in their study revealed the role of two types of cell death, necroptosis and autophagy, in the cardiac remodeling of the left and right ventricles in the late phase of post-infarction heart failure. While necroptosis is known to contribute to the pathogenesis of post-infarction heart failure, the role of autophagy remains unclear. Likewise, linkage between these two cell death modalities has not been sufficiently investigated so far. In their study, necroptotic and autophagic proteins in both heart ventricles were analyzed in failing hearts induced by 60 min left coronary occlusion in adult rats. Heart failure had no effect on the expression of necroptotic proteins (RIP1, RIP3). Phospho-RIP3, acting as a pro-necroptotic signal, was oppositely changed in the left versus right ventricle of failing hearts. Changes in autophagy-related proteins (beclin-1, LC3) indicated a lowered rate of autophagy in the left ventricle and the inhibition of both autophagosome formation and maturation in the right ventricle of failing hearts. In contrast, a marker of executed apoptosis (p89 PARP1 fragment) was increased in the right ventricle only. This study showed a different signaling in heart ventricles of the late phase of post-infarction heart failure, highlighting necroptosis itself rather than its linkage with autophagy in the left ventricle, and apoptosis in the right ventricle.

Another two studies investigated alterations in molecular mechanisms in humans. The study of Amaral et al. [10] aimed to characterize levels of circulating cell-free DNA (cfDNA) in women during healthy pregnancy compared to women who developed gestational hypertension, or preeclampsia. The novel findings of this prospective observational study showed that circulating cfDNA is elevated in pregnant women with hypertensive disorders of pregnancy (HDP, including gestational hypertension and preeclampsia) compared to healthy pregnancies. The authors also proposed a cut-off value of plasma total cfDNA concentration (160 ng/mL) as a sensitive and specific tool for the diagnosis of hypertension in both male and female-bearing pregnancies. Importantly, the results of this study showed that total circulating cfDNA levels might vary in HDP depending on disease severity. The authors proposed that the results of their study may represent a step forward towards the establishment of plasma total cfDNA as a biomarker and pathophysiologically relevant mediator of HDP. Finally, the article by Hsu et al. [11] investigated the association between acrylamide (one of the most common toxins in food) metabolites, N-acetyl-S-(2-carbamoylethyl)-cysteine (AAMA) and N-acetyl-S-(2-carbamoyl-2-hydroxyethyl)-cysteine (GAMA), and cardiovascular risk in 112 children and adolescents with early stages of chronic kidney disease (CKD). The study showed that patients with chronic kidney disease stage G2–G4 had a lower urinary acrylamide level, but a higher AAMA-to-GAMA ratio than those with chronic kidney disease stage G1. Urinary acrylamide level was negatively associated with high systolic and diastolic blood pressure load on 24 h ambulatory blood pressure monitoring. Lower urinary levels of acrylamide, AAMA, and GAMA were correlated with left ventricular mass. Additionally, the study showed that GAMA was superior to AAMA related to nitric oxide-related parameters, namely citrulline and symmetric dimethylarginine. Despite several limitations, the findings of this study suggest that children with chronic kidney disease may have a lower acrylamide conversion capacity or a lower excretion rate, leading to a higher internal exposure, which may increase the risk of developing high blood pressure.

All articles involved in this Special Issue presented new and valuable information on the mechanisms involved in the development and progression of cardiovascular disorders and their prevention and/or treatment. As the Guest Editors of this Special Issue, we would like to acknowledge all authors who contributed either by original experimental studies and clinical studies, or by reviewing recent literature, making this Special Issue valuable for a diverse audience of researchers interested in the investigation of molecular mechanisms associated with cardiovascular disorders as well as in the prevention and treatment of cardiovascular diseases.
